# Early Outcome of Laparotomy Wounds in Pediatric Patients in TASH, Addis Ababa, Ethiopia: A Six-Months Prospective Study

**DOI:** 10.4314/ejhs.v31i1.13

**Published:** 2021-01

**Authors:** Fisseha Temesgen, Abay Gosaye, Nichole Starr, Woubedil Kiflu, Hana Getachew, Belachew Dejene, Amezene Tadesse, Miliard Derbew, Tihitena Negussie

**Affiliations:** 1 Addis Ababa University, Tikur Anbessa Specialized Hospital, Department of Surgery, Zambia St., Addis Ababa, Ethiopia; 2 University of California, San Francisco, Department of Surgery, 505 Parnassus Ave. S-321, San Francisco, CA, 94143

**Keywords:** Pediatrics, SSI, Wound dehiscence, Global Surgery, Ethiopia

## Abstract

**Background:**

Surgical Site Infection (SSI) and wound dehiscence are two early complications of laparotomy causing significant morbidity and mortality. This study was conducted to determine the prevalence and risk factors of SSI and wound dehiscence in pediatric surgical patients.

**Methods:**

We performed a prospective observational study of all pediatric surgical patients who underwent laparotomy at Tikur Anbessa Specialized Hospital, Ethiopia, from December 2017 to May 2018. Data collected included demographics, operative indication, nutritional status, prophylactic antibiotics administration, and duration of operation. Primary outcome was SSI; secondary outcomes were hospital stay and other postoperative complications, including wound dehiscence and mortality. Data were analyzed using SPSS, Version 23. Fisher's exact and Chi-squared tests used to report outcomes. Multivariable logistic regression was used to identify variables associated with SSI, wound dehiscence and other outcomes.

**Results:**

Of 114 patients, median age was 46 months [range: 1day-13 years]; 77(67.5 %) were males. Overall SSI rate was 21.05%. Nine (7.9%) developed wound dehiscence while 3(2.6%) had abdominal contents evisceration. Overall mortality rate was 2.6%. In multivariate analysis, prophylactic antibiotics administration (AOR=13.05, (p=0.006)), duration of procedure (AOR=8.62, (p=0.012)) and wound class (AOR=16.63, (p=0.034)) were independent risk factors for SSI while SSI was an independent predictor of prolonged hospital stay, >1 week (AOR=4.7, p=.003,) and of wound dehiscence (AOR=33. 96, p=0.003). Age (p=0.004) and malnutrition (p<0.001) were significantly associated with wound dehiscence.

**Conclusion:**

SSI and wound dehiscence are common in this setting. Wound contamination, antibiotics administration >1 hour before surgery and operative time >2 hours are independent predictors of SSI.

## Introduction

Laparotomy is among the most commonly performed procedures in pediatric surgery for a variety of diagnoses. Surgical Site Infection (SSI) and wound dehiscence are two early complications of laparotomy wounds and represent a source of morbidity and mortality. Worldwide, SSI is the most common hospital acquired infection in surgical patients (1,2). SSI is defined as an infection that occurs within 30 days after a surgical procedure, or within one year if a foreign body is implanted during the procedure that affects the surgical incision or deeper organ space at the surgical site (2,3).

SSI is associated with the volume of bacterial contamination of the wound, either at the time of surgery or during postoperative wound care (4). SSI doubles the patient's risk of death after surgery (5) and causes patient discomfort. Moreover, wound complications increase healthcare costs (5,6) and prolong hospital stay (7), resulting in significant economic and psychosocial burden both for the patient and the healthcare system. Particularly in developing economies such as Ethiopia, the strain surgical complications place on the already overburdened healthcare system should not be underestimated (8). There is limited literature on SSI and laparotomy wound dehiscence in children. Of the few publications available, most are from high-resource settings. According to WHO, surgical patients in low-resource settings are at higher risk of developing SSI than those in higher-resource settings undergoing similar operations. Compared to the US, where annual SSI incidence ranges from 2–5% (9), reported SSI incidence in Africa ranged from 14.8% in Ethiopia (10), 22.9% in Nigeria (11), to 35.6% in Tanzania (12). Associated factors with SSI in these studies included wound class, emergency procedures, HIV infection, ASA classification and duration of surgery (10,11,12,13,). In high-resource countries, SSI incidence has been reduced by active surveillance systems (8).

Similarly, wound dehiscence is much more common in low resource settings, with incidence in developed countries ranging from 0.2–1.2% (14) compared to 2.1% in Nigeria (15). Associated mortality was 8–45% after wound dehiscence (15) and was associated with emergency surgery, wound class, and postoperative abdominal distension (15).

Understanding the incidence of SSI and wound dehiscence for pediatric populations in low-resource settings and associated risk factors is essential for improving rationale use of antibiotics and other infection prevention practices to improve surgical outcomes. This study was conducted to determine the prevalence and risk factors of SSI and wound dehiscence in children undergoing laparotomy in the pediatric surgery Department at TASH, Addis Ababa, Ethiopia.

## Methods

**Study design and Setting**: This was a prospective observational study conducted in the pediatric surgery Unit at Tikur Anbessa Specialized Hospital (TASH). TASH is the largest referral hospital in Ethiopia and the main teaching hospital for both clinical and preclinical trainings of most disciplines. The study period was from December 1, 2017 to May 31, 2018.

All children who underwent laparotomy in the pediatric surgery unit at TASH from December 1, 2017 to May 31, 2018 were included. Patients referred to TASH with surgical complications but who did not undergo index operation at TASH were excluded. Patient charts were reviewed for data extraction.

After getting informed and signed consent from the parents and care givers, data were collected using a structured questionnaire which included patient demographics, comorbidities, preoperative nutritional status, electrolyte imbalances and anemia, type of operation, wound class, duration of operation, estimated blood loss, perioperative antibiotic administration, length of hospital stay, surgical drains, method of skin closure, suture material used, and any intraoperative complications. Primary outcomes were SSI and wound dehiscence. Secondary outcomes were other postoperative complications including mortality. Patients were followed until discharged from the hospital. Outpatient follow-up at one month was conducted via outpatient clinic visit or phone interview.

**Data analysis**: After data were checked for completeness and consistency, they were analyzed using SPSS version 23. Chi-squared tests were used to report outcomes by patient demographics and risk factors. Fisher's exact test was used when appropriate based on sample size. Multivariable logistic regression was used to test the influence of variables on the outcome. A p-value < .05 was deemed significant.

**Ethics approval**: Addis Ababa University, College of Health Sciences, Department of Surgery Ethical Review Committee has approved the study. The protocol number was DOS/86/20/12; Protocol version number was 01 and version date was November 2016.

## Results

**Figure 1:**
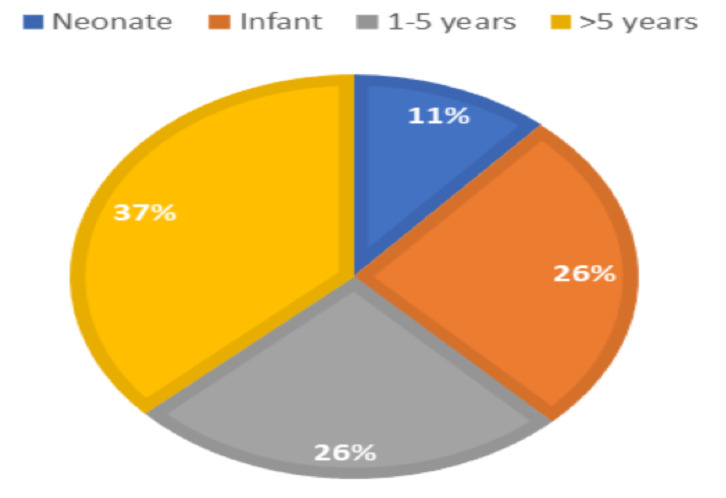
Age distribution

**Patient characteristics and preoperative variables**: A total of 114 patients who had laparotomy were included in the study; 77(67.5%) were males while 37(32.5%) were females. Age ranged from 1 day to 13 years with the most frequent age group in the study was older than five years, 42(36.8%), followed by infants, 30(26.3%), preschool age, 29(25.5%), and neonates, 13(11.4%) (Figure 1). Fifty-one (45%) patients were from Addis Ababa while 63 (55%) were from other parts of the country.

Various urologic disorders, i.e. bladder extrophy, pelvic-ureteric junction obstruction, vesico-ureteral reflux, stone diseases and others make up 22.8% of primary diagnosis. Gastrointestinal abnormalities including hirschsprung disease, 20(17.5%), appendicitis, 13(11.4%), infantile hypertrophic pyloric stenosis (IHPS), 9(7.9%), hepatobiliary disorders, 8(7.0%), abdominal neoplasms 9(7.0%), intussusception 8(7.0%), and anorectal malformations, 7(6.1%) were the major indications for surgery (Table 1).

**Table 1:** Baseline patient and operation characteristics

Characteristics		Count	Percentage
Sex	Male	77	67.5%)
	Female	37	32.5%
Admission diagnosis	Urologic disorders	26	22.8%
	Hirshsprung disease	20	17.5%
	Appendicitis	13	11.4%
	IHPS	9	7.9%
	Intussusceptions	8	7%
	Hepatobiliary disorders	8	7%
	Intraabdominal Neoplasms	8	7%
	Anorectal malformations	7	6.1%
	Other gastrointestinal anomalies	15	13.3%
Nutritional status	Malnourished	26	22.8%
	Well-nourished	88	87.2%
Type of surgery	Emergency	41	36%
	Elective	73	64.0%
Wound class	Clean	23	20.2%
	Clean contaminated	59	51.8%
	Contaminated	22	19.3%
	Dirty	10	8.8%

Twenty-one (18.4%) patients had chronic medical conditions, with 7(6.1%) patients having a malignant condition, and were on preoperative chemotherapy; 6(5.3%) patients had anemia. Cardiac abnormality, chronic renal failure, Diabetes Mellitus (DM) and Down's syndrome constituted the remainder of the medical conditions. Malnutrition was common with 26 (22.8%) patients meeting diagnostic criteria. Among the 114 operations, 73(64.0%) were elective, and the remaining 41(36%) were emergency operations.

All patients were either given preoperative antibiotic prophylaxis or were on scheduled antibiotic treatment already. Ceftriaxone with or without metronidazole was the antibiotic most frequently given. Eighty percent of the patients received antibiotics when they were on the operation table before or after induction of anesthesia. The rest (20%) were given in the ward at least an hour before the operation. The most common wound class was clean-contaminated with 59(51.8%) in this category; 23(20.2%), 22(19.3%) and 10(8.8%) of wounds were clean, contaminated and dirty respectively (Table 1). The SSI rates were 10(8.7%), 12(10.2%), 68(59.1%) and 34(30%) in clean, clean contaminated, contaminated and dirty wounds respectively. The duration of operation was prolonged for two or more hours in 41(36.0%) of patients while 73(64.0%) of the operations lasted for less than two hours. There was no redosing of antibiotics during the procedure.

Seven (6.1%) patients had intraoperative bleeding which was significant and required transfusion. The majority of wounds were closed in two layers (93%) using a continuous running technique for fascial closure. Polyglactic acid (vicryl) of variable thickness was the suture material universally used for fascial and skin closure. The skin closure technique was variable, including sub-cuticular in 70(61.4%) cases, interrupted in 38(33.3%) cases and 6(5.2%) were left open for delayed primary closure. Almost all (95 (83%)) of the operations were performed either by, or in the presence of, a consultant or a fellow.

**Outcomes**: There were 25(21.9%) early wound related complications of which twenty-four (21.05%) patients developed SSI of varying severity. Sixteen (14.04%) were superficial incisional SSI, eight (7.01%) were deep incisional SSI. However, there were no deep space infections reported. Nine (7.9%) patients developed wound dehiscence with 3(2.6%) having complete dehiscence with evisceration requiring emergency reoperation. Out of nine patients who developed dehiscence, 8(88.9%) had SSI, and one (11.1%) patient had clean wound while the other patients, 1(11.1%), 3(33.3%) and 4(44.4%) had dirty, clean contaminated and contaminated wounds respectively. Of those with complete fascial dehiscence, one was a neonate who later died from sepsis, one infant later developed incisional hernia, and the third had complete dehiscence without SSI which might be technical fault.

Postoperative length of stay was considered prolonged if the patients stay seven or more days; 53(46.5%) patients had a prolonged hospital stay. There were three patient deaths making mortality rate 2.6%. Two of the deaths were neonates with generalized peritonitis who died of sepsis with multiorgan failure. The other was an infant with biliary atresia who died on his 40^th^ postoperative day secondary to sepsis following ascending cholangitis.

**Demographic variables and wound complications**: Of the neonates, five (38.5%) had SSI which is a relatively higher rate of surgical site infection compared to infants and older children where children between the age of one and five years had lowest infection rate, but this difference was not statistically significant. The rate of wound dehiscence was also highest in neonates 4(30.8%) while no children older than five years developed wound dehiscence. There was a significant association between age and the development of wound dehiscence (p= .004), but no significant association was observed between age of the patient and development of SSI (p= .346).

**Admission diagnosis**: Of the 24 patients who developed SSI, 10(41.6%) had urologic procedures. The second most common diagnosis was acute abdomen including appendicitis, intussusception and bowel obstruction, with a total of 6 (25%) patients. Hirschsprung disease was the next most common initial diagnosis accounting for 3 (12.5%) of SSI. These three diagnoses also accounted for 88.9% (8 out of the 9) cases with wound dehiscence.

**Antibiotic Administration**: Ceftriaxone alone or in combination with metronidazole was used as the prophylactic antibiotic of choice. The type of antibiotic administered was not associated with any early wound complications. Eight patients (50%) who received antibiotics more than one hour before surgery developed SSI, while only 16(17.2%) of those who received antibiotics during induction of anesthesia (30 minutes before skin incision) developed SSI. Those children who were not given antibiotics during anesthesia induction were already on antibiotic therapy in the wards. Timing of preoperative antibiotic administration was an independent risk factor in predicting surgical site infection (OR adjusted=13.05, P= 0.006). Those children who were already on IV antibiotics treatment before surgery for whom antibiotics were given more than an hour before surgery, for whom prophylactic antibiotics was not given on the operating table, had 13 fold increased odds for development of SSI compared to those children who were given antibiotics during induction (Table 2). However, timing of antibiotic administration was not found to be statistically associated with wound dehiscence.

**Table 2:** Results of univariate and multivariate analysis: Factors associated with SSI\

Variables	Univariate (P-value)	Multivariate (P-value, OR adjusted)
**Age of the patient**	0.346	0.440
**Nutritional status**	0.001	0.997
Timing of prophylactic antibiotics administration*	**0.004**	**0.006, [13.05]**
Duration of the procedure*	**0.007**	**0.012, [8.62]**
**Method of skin closure**	0.283	0.107
Wound class [contaminated wound]*	**0.036**	**0.034, [16.63]**

**Nutritional status**: Children were categorized as well-nourished and malnourished based on nutritional status (Appendix I). Malnutrition ranged from simple underweight to severe acute malnutrition and was present in 26(22.8%) patients, while 88(77.2%) were well nourished. There was no child with obesity. Malnutrition was significantly associated with both SSI (p=0.001) and wound dehiscence (p<0.001).

**Surgical wound class and surgical techniques**: Surgical site infection developed in 2(8.7%) of clean wounds, 6(10.2%) of clean contaminated wounds, 13(59.1%) of contaminated wounds, and 3(30%) of dirty wounds. Wound class was found to be an independent risk factor for SSI (OR adjusted=16.63, P=0.034) (Table 2). There was similarly higher rate of wound dehiscence in contaminated, 4(18.2%), and dirty, 1(10%), wounds, but the observation was not significant, (p=0.23).

Duration of operation greater than two hours was found to be an independent predictor of development of SSI (OR adjusted =8.62, P=0.012) although operation time was not found to be significantly associated with wound dehiscence.

Wound closure was performed using three different techniques: subcuticular, interrupted or delayed primary wound closure. The method of skin closure was not significantly associated with SSI (p= .283) though associated with wound dehiscence (p= .025).

**Factors associated with SSI and wound dehiscence**: Univariate logistic regression analyses were performed for all variables as potential predictors of SSI and wound dehiscence. All variables selected as potential predictors of SSI and wound dehiscence were included in multivariate analysis. These included age of the patient, nutritional status, timing of prophylactic antibiotics administration, duration of the procedure, wound class and method of skin closure (Table 2). In multivariate analysis, timing of prophylactic antibiotics administration (OR adjusted= 13.05, p=0.006), duration of the procedure (OR adjusted= 8.62, p=0.012) and wound class (OR adjusted= 16.63, p=0.034) were found to be independent risk factors for SSI but not for wound dehiscence (Table 2), whereas Age (p= .004), malnutrition (p<.001) and method of skin closure (p= .025) were significantly associated with wound dehiscence and SSI was an independent predictor of wound dehiscence (OR adjusted=33. 96, p=.003) (Table 3).

**Table 3:** Results of univariate and multivariate analysis: Factors associated with wound dehiscence

Variables	Univariate (P-value)	Multivariate (P-value, OR adjusted)
**Age of the patient**	0.004	0.079
**Nutritional status**	< 0.001	0.999
**Timing of prophylactic antibiotics** **administration**	0.644	0.186
**Duration of the procedure**	0.279	0.396
**Method of skin closure**	0.025	0.280
**Wound class [contaminated wound]**	0.231	0.621
Wound infection (SSI)*	**<0.001**	**0.003 [33]**

Eight of the nine patients (88.9%) who had wound dehiscence also had SSI, including the three patients who had complete dehiscence with evisceration. Surgical site infection was found to be significantly associated with the development of wound dehiscence (p< .001) and an independent predictor of wound dehiscence (OR adjusted=33. 96, p=.003) (Table 3).

Presence of SSI was also found to be an independent predictor of prolonged hospital stay greater of than 7 days (OR adjusted=4.7, p=.003), and wound dehiscence conferred 10 times the odds of prolonged hospital stay (>7 days) compared to those with no dehiscence (AOR=10.67, p=.028)

There was no statistically significant association between patient sex, place of residence (Addis Ababa vs. out of Addis Ababa), duration of preoperative hospital stay, chronic medical illness, preoperative anemia, type of operation (emergency vs. elective), type of antibiotics used, level of operating surgeon, and layers of wound closure with SSI or wound dehiscence.

## Discussion

In our study, overall SSI rate was found to be 21.05%. This figure is far higher than the reports seen in developed countries like in United States which showed SSI prevalence of 0.99% though it was conducted on pediatric general, orthopedic, and plastic surgical patients (16). A similar study done in Italy including all pediatric surgical procedures showed the rate of SSI to be 1.0% (17). As both studies involved other surgical procedures in addition to laparotomy, these populations may see relatively reduced incidence of SSI as compared to abdominal operations which are more likely to experience infectious complications due to contamination. Some studies showed that SSI incidence was significantly lower after ear, nose and throat procedures compared to all other procedures (18), but this alone cannot explain the wide difference between the reports. WHO's report also supports this finding, concluding that the risk of SSI in developing countries, particularly in sub-Saharan Africa, is higher than that in high-resource settings who undergo similar procedures (18,19). A study from Benin city reported SSI rate of 11.8% in neonatal surgical operation (20). Similarly a study done in Nigeria showed SSI rate in children undergoing any surgical operation to be 23.6% (11), and abdominal SSI incidence in children was 22.9 % (13). A previous study done in Ethiopia in 2005 showed the SSI rate to be 14.8% (10), however this study included all pediatric and adult surgical patients at TASH. The infection rate in our study is very similar to the reports from these African countries.

In this patient population, malnutrition, wound contamination, antibiotics administration at least one hour before surgery and prolonged duration of the procedure more than 2 hours were found to be risk factors for SSI while the last three risk factors are also independent predictors for development of SSI. In the study done by Butcher et al., wound classification and antibiotic administration were not independent predictors of SSI, but age of the patient was an independent predictor of SSI whereas our study did not find this to be a significant risk factor (16). Our findings are consistent with those in prior Italian studies, where duration of surgery was a risk factor for SSI (17), as well as with the degree of incisional contamination and a long duration of surgery (>2 hrs.) seen in a Nigerian study (11).

In our study, 7.9% of patients developed wound dehiscence while 2.6% had complete fascial dehiscence with abdominal evisceration. This rate is comparable with a previous study in the same center on relaparotomy in pediatrics showing that seven out of 354(2%) developed complete wound dehiscence and accounted for the 13.2 % indication of relaparotomy (21). In the limited literature on this topic, reported incidence in developed countries ranges between 0.2–1.2 percent (14) whereas one report from Nigeria showed a complete fascial dehiscence prevalence of 2.1% (20). Age (Neonate), malnutrition and SSI were found to be a risk factors for wound dehiscence in our study. This is consistent with findings in Benin City, Nigeria, where age was a significant predictor of wound dehiscence (20). Our findings are also consistent with a study done in Amsterdam, the Netherlands, which showed age <1 year and wound infection to be an independent risk factors for wound dehiscence (14), although in our study, only SSI (wound infection) was found to be an independent predictor of wound dehiscence.

Our study did not include isolation of the offending microorganisms and did not study antibiotics susceptibility profile. This is an evaluation of one hospital and our findings may not apply to other settings or countries. This is a tertiary referral center, so our findings may not reflect SSI burden in more rural or less resourced settings.

In conclusion, the rate of SSI and wound dehiscence is high in our pediatric surgical population at TASH. Wound contamination, antibiotics administration more than an hour before surgery and prolonged duration of the procedure more than 2 hours are independent predictors for development of SSI. Age, malnutrition and SSI were also found to be a risk factors for wound dehiscence. While some of these patient factors are difficult to address in the short term such as presenting diagnosis leading to wound contamination, and malnutrition, other factors such as appropriate administration of prophylactic antibiotics are potentially modifiable. Future focus of the department in terms of prevention measures could be ensuring preoperative antibiotic administration within 60 minutes of incision as per the WHO Surgical Safety Checklist to be used routinely, as well as efforts to reduce duration of procedures. Local antibiotic use surveillance study should also be done to know the most common offending microorganisms and their antimicrobial susceptibility. In some elective or semi-elective cases, preoperative nutritional supplementation may also be a possible mechanism by which to reduce infections and wound complications.
